# Analysis of the Oxidative Burst and Its Relevant Signaling Pathways in *Leptosphaeria maculans—Brassica napus* Pathosystem

**DOI:** 10.3390/ijms22094812

**Published:** 2021-05-01

**Authors:** Cunchun Yang, W. G. Dilantha Fernando

**Affiliations:** Department of Plant Science, Faculty of Agricultural and Food Sciences, University of Manitoba, Winnipeg, MB R3T 2N2, Canada; umyang48@myumanitoba.ca

**Keywords:** *Leptosphaeria maculans*, *Brassica napus*, reactive oxygen species (ROS), hydrogen peroxide (H_2_O_2_), programmed cell death (PCD), respiratory burst oxidase (RBOH)

## Abstract

An oxidative burst is an early response of plants to various biotic/abiotic stresses. In plant-microbe interactions, the plant body can induce oxidative burst to activate various defense mechanisms to combat phytopathogens. A localized oxidative burst is also one of the typical behaviors during hypersensitive response (HR) caused by gene-for-gene interaction. In this study, the occurrence of oxidative burst and its signaling pathways was studied from different levels of disease severity (i.e., susceptible, intermediate, and resistant) in the *B. napus*–*L. maculans* pathosystem. Canola cotyledons with distinct levels of resistance exhibited differential regulation of the genes involved in reactive oxygen species (ROS) accumulation and responses. Histochemical assays were carried out to understand the patterns of H_2_O_2_ accumulation and cell death. Intermediate and resistant genotypes exhibited earlier accumulation of H_2_O_2_ and emergence of cell death around the inoculation origins. The observations also suggested that the cotyledons with stronger resistance were able to form a protective region of intensive oxidative bursts between the areas with and without hyphal intrusions to block further fungal advancement to the uninfected regions. The qPCR analysis suggested that different onset patterns of some marker genes in ROS accumulation/programmed cell death (PCD) such as *RBOHD*, *MPK3* were associated with distinct levels of resistance from *B. napus* cultivars against *L. maculans*. The observations and datasets from this article indicated the distinct differences in ROS-related cellular behaviors and signaling between compatible and incompatible interactions.

## 1. Introduction

Canola, as a brand of cultivars from rapeseed and field mustard, has become a very important crop to Canada as well as to the world, since they have low amounts of erucic acid and glucosinolate, which have bad tastes and harmful effects on human and animal consumption [1,2]. Blackleg (caused by fungus *Leptosphaeria maculans*) has become such a great threat since 1975 in Canada [3] that it may cause a 50% reduction in the production of canola. The major symptoms caused by the pathogen include stem canker, root rot, leaf lesion (with pycnidia), and pycnidia and pseudothecia on stubble [4].

Traditionally, the blackleg disease is managed by crop rotation, fungicide, etc., however, the development of genetically resistant rapeseed (*Brassica napus*) is the most cost-effective and environmentally friendly strategy for dealing with the disease [5]. *L. maculans* possesses various enzymes to reinforce its infection upon hosts. Three cell-wall-degrading enzymes (CWDEs): endopolygalacturonase (*pg1*) and two cellulases (*cel1* and *2*), and *cel2* transcripts are found in the cotyledons and leaves from *B. napus* and *B. juncea* [6]. Moreover, *L. mauclans* also secretes phytotoxins such as sirodesmin PL, which causes leaf lesion [7,8].

Generally, there are two types of disease resistance in plants (triggered by phytopathogenic infection): qualitative and quantitative resistance. Qualitative resistance is triggered by gene-for-gene interactions, and it represents one type of interaction between the Avr proteins from the pathogens (AvrLm for *L. maculans*) and R proteins from the hosts (Rlm for *B. napus*). The interaction induces a hypersensitive response (HR) which triggers a series of rapid localized signaling cascades including ROS production, programmed cell death (PCD), and systemic acquired resistance (SAR). On the other hand, quantitative resistance exhibits a slower but more persistent defense in which more complicated internal signaling networks are involved [9,10,11].

Oxidative burst including reactive oxygen species (ROS) production is an early response to various biotic/abiotic stresses in plants, which is considered a crucial part of the defense against biotic/abiotic stresses [10,12,13]. During the plant defense, ROS accumulation, and the following signaling cascades exert various defense mechanisms that halt the pathogen invasion [10]. Those mechanisms include electrolyte leakage, modification of plant cells, programmed cell death (PCD), hormonal signaling, and protein production [10,12,14,15,16].

Various studies correlated the electrolyte leakage and ROS-responsive defense activities. Electrolyte leakage has been found potentially connected with PCD and ROS generation/signaling [17,18,19,20]. Localized H_2_O_2_ secretion is the early response of HR from the origins of infection, including cell wall cross-linking and membrane damage [21,22]. Apoplastic peroxidases (such as peroxidases 33 and 34) are also involved in the PAMP Triggered Immunity (PTI) against plant pathogens [23,24], the binding between PAMP molecules and receptor-like R protein results in the activation of ROS-related factors including RBOHs (Respiratory Burst Oxidase Proteins), MAPK (Mitogen-Activated Protein Kinase) signaling and Ca^2+^ transportation [15,25]. Moon et al., (2003) [26], suggested that the two MAPK cascade factors, *MPK3*, and *6* are activated by ectopic H_2_O_2_ accumulation. Furthermore, a gene named *ETHYLENE RESPONSE FACTOR6* (*ERF6*) was activated by MPK3/6 cascade to induce *PDF1.1* and *PDF1.2,* which enhance plant defense in Arabidopsis [27]. Wang et al., (2009) [28] suggested that *MPK4* suppresses the ROS production in *Brassica napus*, and *MPK4* was found to induce jasmonic acid (JA) induced *PDF1.2*; the overexpression of *MPK4* connected with enhanced resistance against a necrotrophic pathogen, *Sclerotinia sclerotiorum*.

Evidence also shows that hormones such as salicylic acid also respond to oxidative burst. Salicylic acid (SA) and ethylene (ET) secretion respond to oxidative burst to elicit cellular signals towards lesion extension (i.e., programmed cell death), while JA responsive factors played the opposite roles [19,29]. According to Overmyer et al. (2000) [19], ethylene (ET) has its dependent pathway to induce cell death ahead of lesion formation before the symptoms emerge, and this process was activated by superoxide, JA response factors such as *JAR1* played a negative role in superoxide/ET-induced cell death.

The interaction between R and Avr proteins leads to hypersensitive response (HR), which involves various defense mechanisms including Ca^2+^ signaling and MAPK signaling, localized cell death (LCD) to hinder further pathogenic progression [12,16,25,30]. Studies have shown that HR triggers a set of defense mechanisms that are similar to those from basal resistance, while the signal transduction is activated earlier and more localized [16,22,31], and the subsequent cell-cell communication sends the defense signals towards adjacent plant cells using ROS molecules as the messenger [15,16,21,22,31].

Both basal resistance and gene-for-gene interaction utilize ROS accumulation to combat pathogenic invasion, these two types of resistance also represent two types of the genetic background of *B. napus,* which the plant breeders have been attempting to breed for efficient blackleg resistance. Therefore, it is necessary to have a deeper understanding of ROS responsive signaling pathways.

The goal of the study is to describe how ROS production and signaling function in the *Brassica napus*–*Leptosphaeria maculans* interaction, we intended to elucidate the role of ROS signaling with different severities of *B. napus* defense. By analyzing and comparing the pivotal genes within ROS signaling pathways, the onset patterns and expression levels of those studied genes can explain the various disease severities among different canola cultivars. The observations of cytological behaviors were also able to visualize the effects of ROS signaling on disease resistance.

## 2. Results

### 2.1. Early Induction of Electrolyte leakage Occurring from Intermediate and Resistant Phenotypes

**Figure 1:**
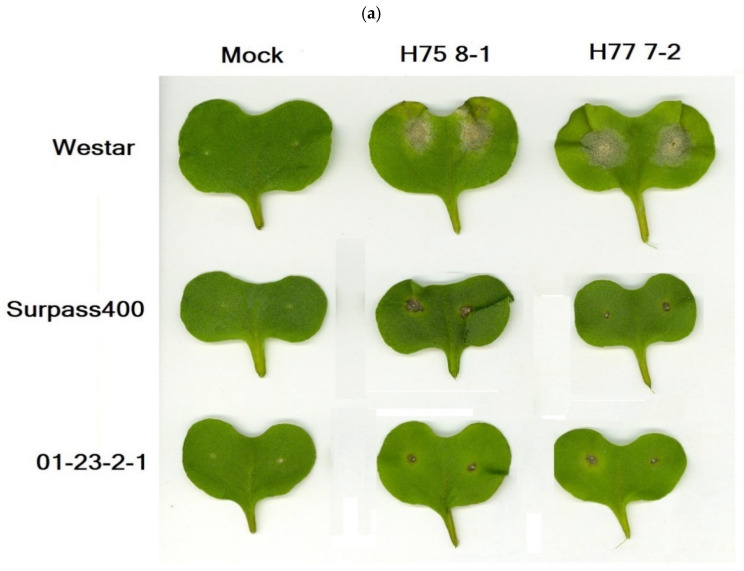

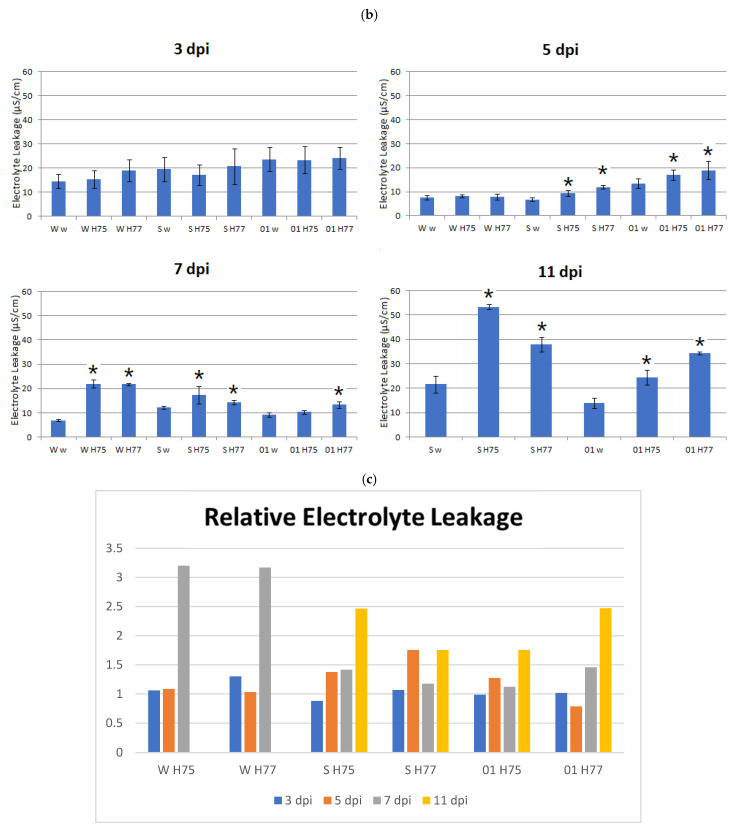
(**a**) The lesion development and the appearance of phenotypes were observed from the three genotypes (Westar, Surpass400 and 01-23-2-1) and two isolates (H75 8-1 and H77 7-2) at 11 days post-inoculation (dpi). (**b**) The measurement of electrolyte leakage at 3, 5, 7, and 11 dpi. At 11 dpi, because Westar cotyledons were generally degraded and heavily infected, the measurement of electrolyte leakage at this stage became incapable and inaccurate, the measurement of Westar genotype at this time point was not included. The x-axis indicates the inoculation pair between genotypes (W: Westar; S: Surpass400; 01: 01-23-2-1) and isolates (w; water; H75: H75 8-1; H77: H77 7-2), the y-axis indicates the voltage detected from the cotyledon-soaked solution suggesting the leaking of ions (unit: µS/cm, S; Siemens). The asterisks indicate the significant differences of the electrolyte leakage measurement among mean values when compared with mock inoculation (Fisher’s Least Significant Difference; *p* < 0.05). (**c**) The relative electrolyte leakage at 3, 5, 7, and 11 dpi. The relative leakage is calculated by dividing the average measurements of inoculated cotyledons by mock inoculated ones. For time point, different lowercase letters suggest the significant differences among mean values (Fisher’s Least Significant Difference; *p* < 0.05). The results are based on three replicates in three independent experiments.

As shown in Figure 1, two selected *L. maculans* isolates (HCRT75 8-1 and HCRT77 7-2) produced three distinct levels of severity on three *B. napus* cultivars (Westar, Surpass400 (*BLMR1/LepR3* and *BLMR2/RlmS*), and 01-23-2-1 (*Rlm7*)). The inoculation caused susceptible phenotypes on Westar cotyledons; Surpass400 and H75 8-1 had intermediate incompatible interaction (AvrLepR2–BLMR2) while Surpass400–H77 7-2 (AvrLm1–BLMR1), 01-23-2-1–H75 8-1/H77 7-2 (AvrLm 4-7–Rlm7), these three cases showed strong incompatible (resistant) interaction [32,33]. The differences in severity reflected the distinct modulation of defense signaling in those cultivars, and the study of these differences helps explain how susceptibility and resistance occur in canola when combating the blackleg pathogen.

To understand how oxidative burst works at a physiological level, the measurement of electrolyte leakage is a useful tool. As one of the earliest responses to various stresses, electrolyte leakage is found to trigger multiple defensive mechanisms in planta, which includes hormonal secretion, programmed cell death, oxidative burst, etc. [17,19,20]. For this study, the electrolyte leakage was measured from the excised cotyledons, the voltage caused by the leaked electrolytes from both mock and inoculated samples were measured with the VWR sympHony conductivity meter.

As shown in Figure 1, the two resistant genotypes Surpass400 and 01-23-2-1 exhibited a significantly higher level of electrolyte leakage (compared with mock-inoculated cotyledons) as early as 5 dpi, when the susceptible Westar cotyledons did not have the induction of significant electrolyte leakage. Westar started to induce higher leakage at 7 dpi, and at 11 dpi, the cotyledons were collapsed and severely damaged to perform a further measurement, therefore, there was no data about Westar leakage at 11 dpi. Surprisingly, Surpass400–H75 8-1 seemed to have retained the secretion of electrolyte at 11 dpi according to the conductivity measurement. The results suggested that resistant genotypes had earlier activation of electrolyte secretion (at 5 dpi) while the compatible interaction (i.e., Westar) had a later triggering process (at 7 dpi).

### 2.2. Distinct Detection of Hydrogen Peroxide in Susceptible, Intermediate and Resistant B. napus Plants

As a stable and reactive ROS molecule, H_2_O_2_ plays multiple roles in plants during normal physiological functioning and stress resistance, its membrane-permeable property makes it a useful messenger in cell-cell communication, thus coordinates cellular signaling mechanisms which are time/space-dependent [12,14,34]. By 3,3′–Diaminobenzidine (DAB) staining, the diffusion of hydrogen peroxide (H_2_O_2_) was visualized as brown-colored stains.

**Figure 2:**
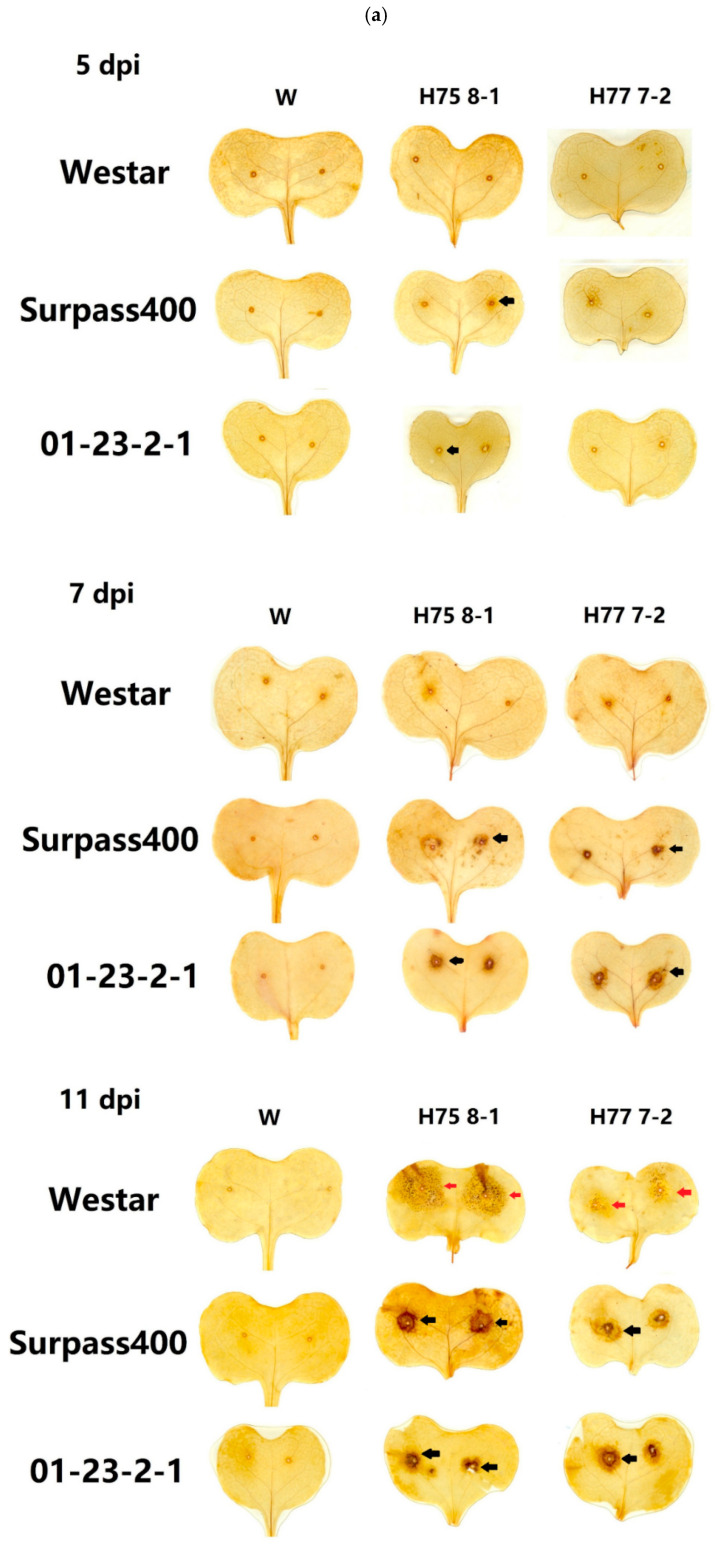

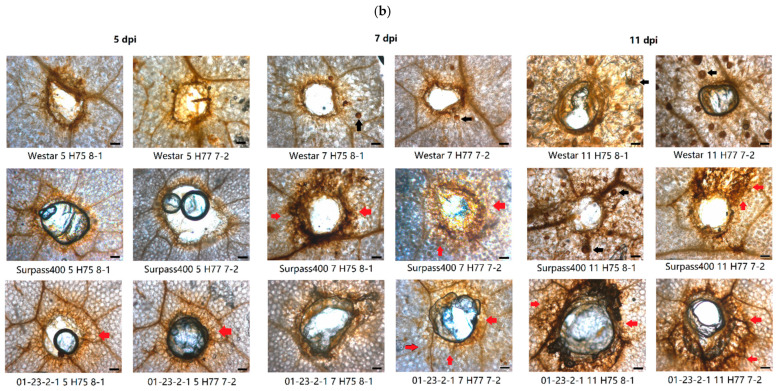
In situ detection of hydrogen peroxide by DAB staining on cotyledons of Westar/Surpass400/01-23-2-1 inoculated with H75 8-1/H77 7-2 (genotype/isolate). (**a**) The scanned cotyledon images (representative images) of stained cotyledons displayed the diffusion of ROS molecule H_2_O_2_ after inoculation (with mock, H75 8-1 and H77 7-2). The brownish stains (black arrows) suggested the diffusion regions of H_2_O_2_. Westar at 11 dpi also showed some pycnidia production (red arrows). (**b**). The representative microscopic images taken from the origins of inoculation (magnitude: 50×), the images showed the details about H_2_O_2_ accumulation when the fungus progressed from the puncture holes for inoculation. The brownish color shown from Surpas400 and 01-23-2-1 had more captured H_2_O_2_ around the origins of inoculation (red arrows) and some pycnidia from Westar and Surpass400 were also captured (black arrows). The microscopic images were taken at 5, 7, and 11 days post-inoculation (dpi). Bars = 100 µm.

As shown in Figure 2, at 5 dpi, it is difficult to compare/contrast the patterns of H_2_O_2_ among the six inoculation treatments. At 7 and 11 dpi, both the intermediate and resistant genotypes Surpass400 and 01-23-2-1 exhibited more captured brownish color, formed a ring-like pattern surrounding of the origins of inoculation (Figure 2a, red arrows). On the other hand, Westar cotyledons had no intense brownish color around inoculation sites and the pycnidia were visible at 7 dpi.

The microscopic observation revealed a similar pattern of H_2_O_2_ (Figure 2b red arrows). The localized secretion of H_2_O_2_ was visible as early as 5 dpi under the microscope from the cotyledons,01-23-2-1, which displayed some detected brownish (i.e., H_2_O_2_) distribution around the punctured holes (Figure 2). At 7 dpi and 11 dpi, Westar samples (both H75 8-1 and H77 7-2) had a large amount of pycnidia (Figure 2b, black arrows), while Surpass400–H75 8-1/H77 7-2 and 01-23-2-1- H75 8-1/H77 7-2 cotyledons showed an apparent trace of H_2_O_2_ accumulation on the cotyledonary tissues (Figure 2b, red arrows). Adequate H_2_O_2_ accumulation induces considerable signaling, which triggers defense responses at the cellular level, such as MAPK cascades and Ca^2+^ signaling [14,16,35]. Since the accumulation of H_2_O_2_ plays central roles in the activation of plant defense signaling, the intense accumulation of H_2_O_2_ on Surpass400 (7 dpi) and 01-23-2-1 (5 and 7 dpi) cotyledons indicated that the gene-for-gene interaction (for both intermediate and resistant cases) can induce early H_2_O_2_ outburst to trigger anticipated and localized defense activities to inhibit fungal development.

### 2.3. The Impacts of ROS Upon Cell Death

Followed by H_2_O_2_, various physiological activities can be triggered to stop further pathogenic progression, those activities include callose deposition, and cell wall cross-linking [11,12,14,24,36]. Another biological process highly regulated by H_2_O_2_ is the programmed cell death (PCD) [11,12,16].

**Figure 3:**
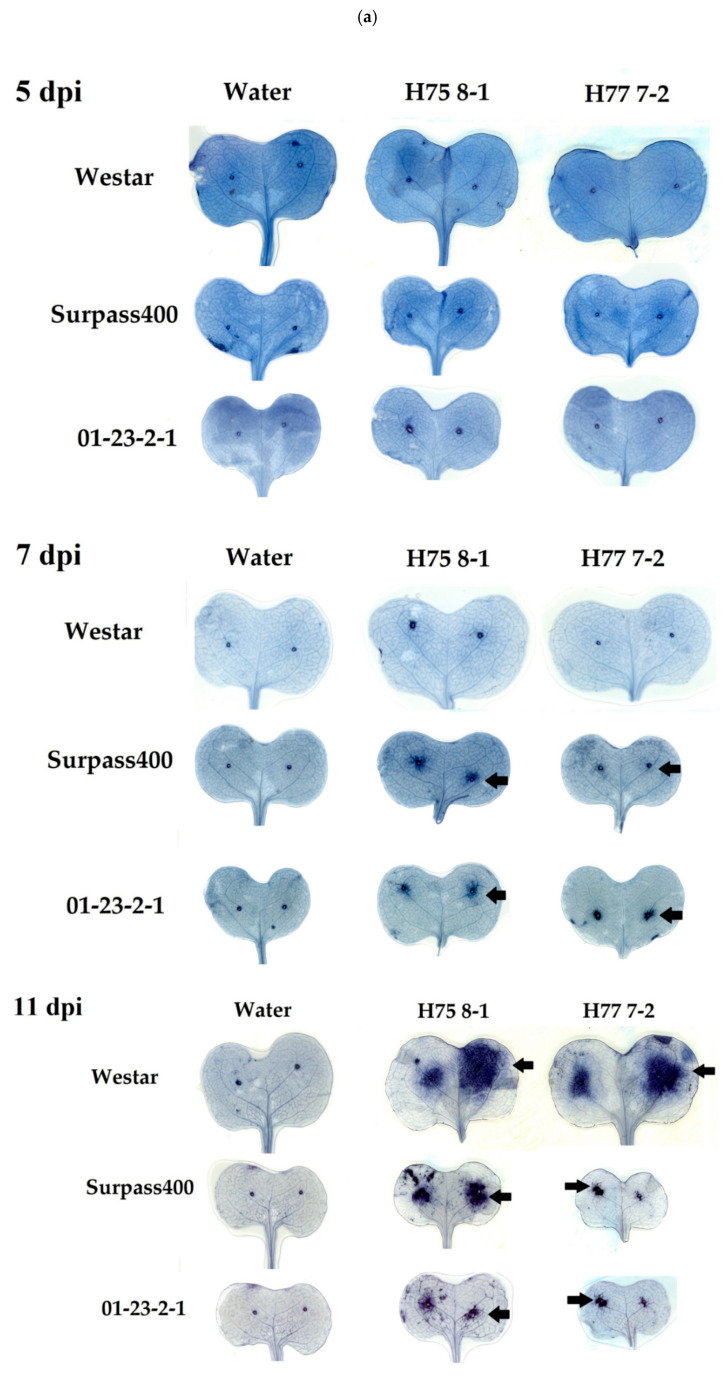

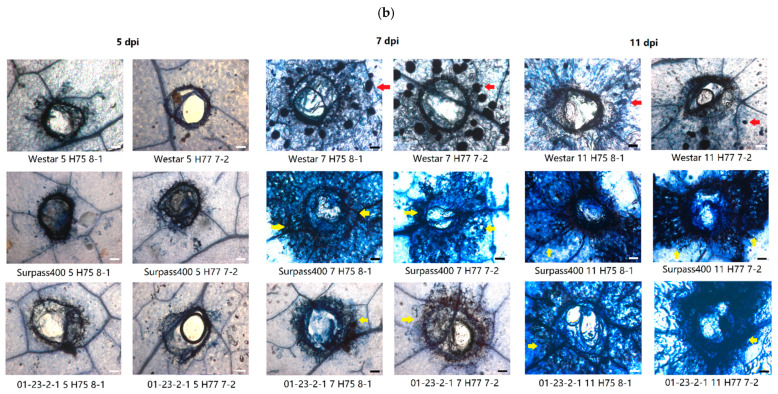
Regions of cell death (stained with trypan blue) at 5, 7, and 11 dpi adjacent to the origin of fungal development from 6 inoculation pairs (genotype/isolate: Westar-H75 8-1/H77 7-2, Surpass400 – H75 8-1/H77 7-2 and 01-23-2-1 – H75 8-1/H77 7-2). (**a**) The scanned cotyledons stained with trypan blue (representative images) showed the spread of cell death (dark blue) throughout the cotyledons initiated from the sites of inoculation (center of each lobe). The potential regions of cell death on the cotyledons were highlighted by black arrows. (**b**) The representative microscopic images (magnitude: 50×) taken around the sites of inoculation, the formations of senescent cells (yellow arrows), hyphal development (red arrows), and pycnidia (red arrows) formations were visualized under the microscope. Bars (black and white) = 100 µm.

By observing the cotyledons treated with trypan blue staining (TBS), the bulk of stained senescent cells were visible from both Surpass400 and 01-23-2-1 cotyledons around the origins of inoculation at 7 dpi (Figure 3a,b). At 11 dpi, Surpass400 and 01-23-2-1 had a further enlargement of death regions, which was an enhanced situation to what happened at 7 dpi. From the microscopic images, the incompatible interaction did not hinder the hyphal formation of *L. maculans* fungus but formed a buffering zone with dead cells (Figure 3a, black arrows, Figure 3b, yellow arrows) to inhibit the chance for hyphae to invade more living tissues for nutrition. On the other hand, Westar only had hyphae (5 dpi) and pycnidia (7 and 11 dpi) formed around the punctured holes for inoculation (Figure 3b, red arrows), suggesting that the LCD was not observed in compatible interaction and this defense mechanism must be the feature for incompatible interaction (HR cell death) (Figure 3b).

### 2.4. Signal Allocation Patterns in ROS Production and Subsequent Responsive Factors among Susceptible, Intermediate and Resistant B. napus Plants

Triggered by oxidative burst, the plant body can trigger a series of defensive mechanisms including expression of responsive genes in hindering further pathogenic progression. These defensive mechanisms include the early apoplastic accumulation of ROS by membrane-bound NADPH oxidases [11].

**Figure 4:**
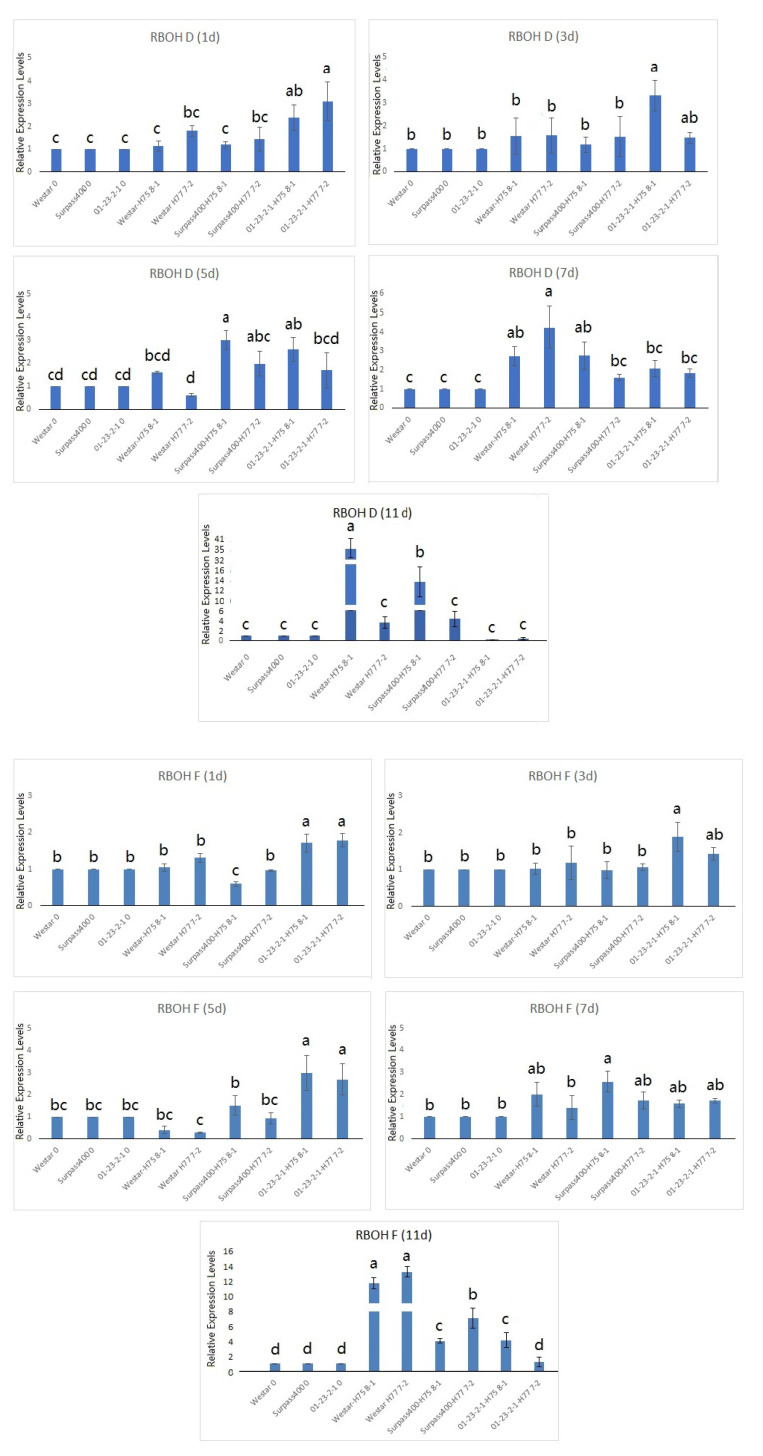

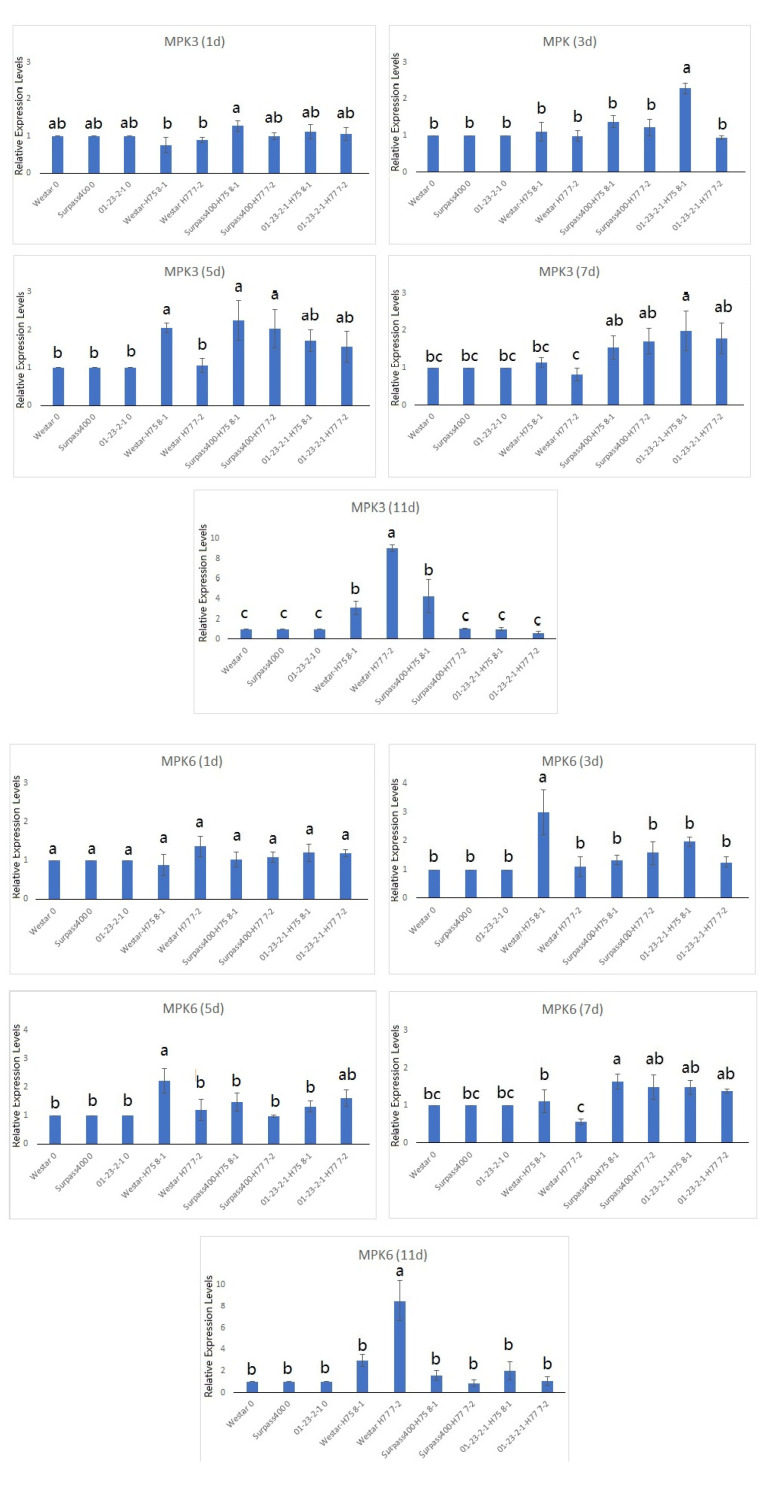
Gene expression of genes related to ROS production (*RBOHD* and *F*) and ROS signaling (*MPK3* and *6*). The level of the bars are the expression levels from the inoculated cotyledons (three genotypes: Westar, Surpass400 and 01-23-2-1, two isolates: H75 8-1 and H77 7-2) normalized with the cotyledons inoculated with water (assuming that the expression of each studied gene in the cotyledons inoculated with water is 1). For time point, different lowercase letters suggest the significant differences among mean values (Fisher’s Least Significant Difference; *p* < 0.05). The results are based on three replicates in three independent experiments.

As shown in Figure 4, Surpass400 and 01-23-2-1 exhibited an earlier induction of *RBOHD* and *F* compared with Westar. 01-23-2-1 showed the relatively high expression of *RBOHD/F* as early as 1 dpi. Surpass400 (both H75 8-1 and H77 7-2) did not show straightforward early induction of both *RBOHD* and *F* (1, 3 and 5 dpi). Remarkably, both Surpass400 H75 8-1 and H77 7-2 showed higher expression *RBOHD* at 5 dpi compared with Westar (Surpass400–H77 7-2 is not significant enough). For Westar, both genes were not expressed until 7 dpi and displayed a high expression level at 11 dpi. As early as 3 dpi, the blackleg fungus started to secrete cell wall degrading proteins (CWDBs) in *B. napus* [37]. Becker et al., (2017) [31] also found the early cell collapse in resistant *B. napus* (incompatible interaction against *L. maculans*) at 3 dpi, and at the same time point, genes related to SA and JA signals are also induced. The results indicated that RBOH enzyme may be an important factor to initiate ROS production during plant defense in *B. napus* since early defense against *L. maculans* seems to be one of the features for effective defense.

**Figure 5:**
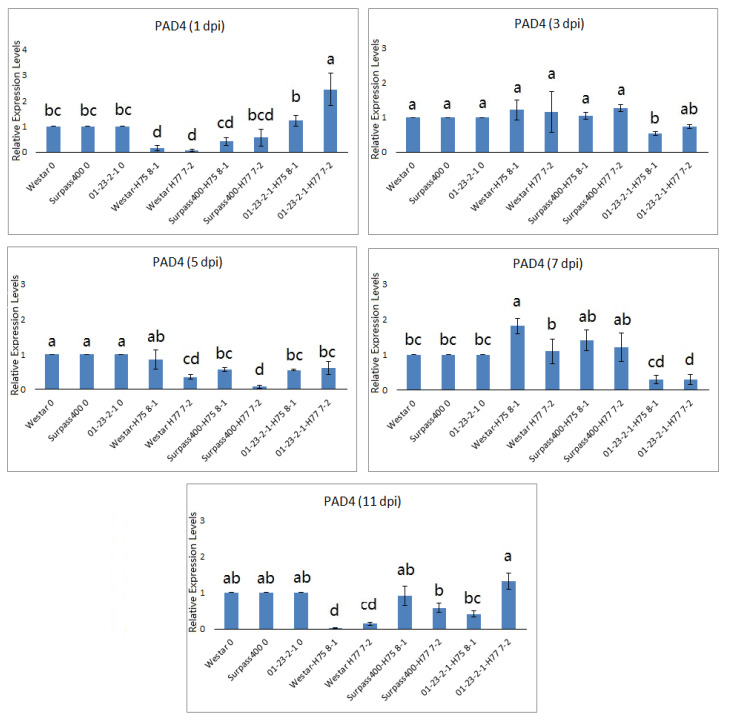

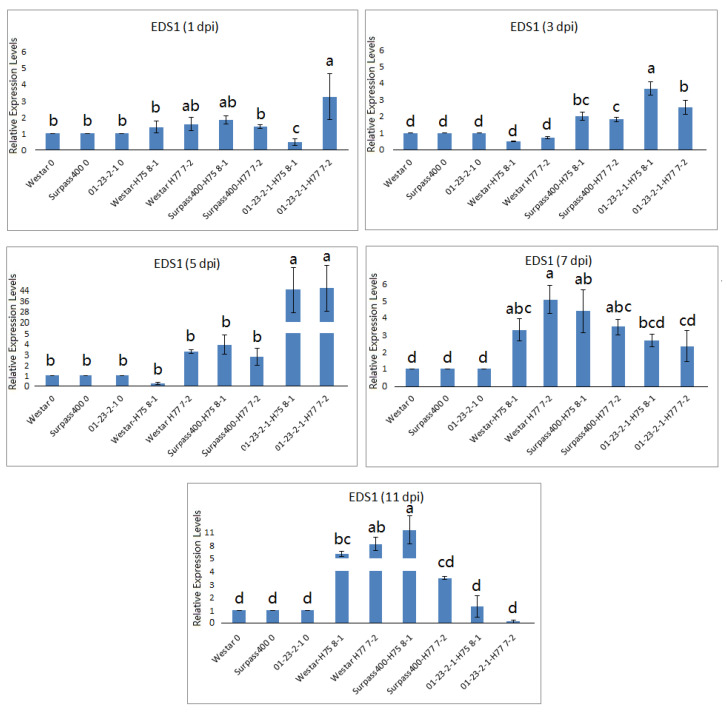
Expression analysis of genes related to cell death (*PAD4* and *EDS1*). The level of the bars are the expression levels from the inoculated cotyledons (three genotypes: Westar, Surpass400 and 01-23-2-1, two isolates: H75 8-1 and H77 7-2) normalized with the cotyledons inoculated with water (assuming that the expression of each studied gene in the cotyledons inoculated with water is 1). For the time point, different lowercase letters suggest the significant differences among mean values (Fisher’s Least Significant Difference; *p* < 0.05). The results are based on three replicates in three independent experiments.

Since PCD is one of the mechanisms of HR defense, the pathogen is not able to get enough nutrients to replicate when it is surrounded by dead cells [16,36,37]. *ENHANCED DISEASE SUSCEPTIBILITY 1* (*EDS1*) and *PHYTOALEXIN DEFICIENT 4* (*PAD4*) are found to play pivotal roles in *R* gene-mediated signaling of resistance [2,36]. By analyzing the expression of *EDS1* (Figure 5), 01-23-2-1 (inoculated with H75 8-1 and H77 7-2) displayed higher expression at 3 and 5 dpi while Westar (with H75 8-1 and H77 7-2) had the peak expression at 11 dpi. Intermediate interaction for cotyledons of Surpass400 with H75 8-1 had early induction of the same gene at 3 dpi, and also exhibited up-regulation at 11 dpi like Westar. The onset patterns of *EDS1* expression suggested that resistant interaction had earlier activation of *EDS1*, possibly due to the earlier recognition of the pathogen by gene-for-gene interaction.

Surprisingly, *PAD4* did not show co-expression with *EDS1*. According to other studies, PAD4 and EDS1 interact with each other to trigger basal resistance and HR [38,39]. *PAD4* did not have a high expression at 5 dpi in Surpass400 and 01-23-2-1 when expression of *EDS1* peaked at this time point in these two genotypes. EDS1 can induce other resistant activities without PAD4 [38,39] and EDS1 is found to bind multiple factors in plant defense [40].

MPK3 and MPK6 are also found to support ROS signaling, these two factors also assist the production of camalexin and ethylene [27,41]. An ethylene-responsive factor, *ERF6*, was phosphorylated and activated by MPK3/MPK6 cascade to induce *WRKY33*. *PDF1.1* and *PDF1.2*, two defensins to enhance plant defense, were also activated [42]. *MPK3* and *MPK6* displayed induction from Westar at 11 dpi (with both HCRT75 8-1 and HCRT77 7-2), while Surpass400 and 01-23-2-1 did not show very high expression (Figure 5). Westar depends more on the expression of *MPK3* and *MPK6* expression levels at a later period (necrotrophic) of the infection, when the fungal progression was too severe that the host needed massive ROS signaling and other defense activities to stop further infection. The high expression of *MPK3* and *MPK6* on Westar cotyledons at 11 dpi also linked to the expression of *RBOHD* and *-F*, suggesting that susceptible Westar cotyledons lately activated massive ROS signaling to stop the necrotrophic phase of *L. maculans*, the ROS molecules are able to exert multiple factors and signaling pathways to activate plant defense activities [15,20].

## 3. Discussion

In this article, the genotypes with stronger resistance Surpass400 and 01-23-2-1 exhibited earlier emergence of electrolyte leakage, H_2_O_2_ diffusion and cell death, compared with susceptible control Westar. Moreover, ROS-responsive genes such as *RBOHD/F* tended to be activated from 01-23-2-1.

Electrolyte leakage has been found in many studies as the early physiological signal for stress response. It is also observed from plant tissues during hypersensitive response and cell death [18,19]. Ions such as K^+^ and Ca^2+^ are transported via ion channels to induce signals related to stress tolerance. The efflux of K^+^ is found in various biological processes including PCD, ROS, stomata closure, and hormonal secretion [17,43]. Besides, another remarkable electrolyte Ca^2+^ is originated from the vacuole and induced as the second signal when the MAMP/DAMP (Microbe/Damage-Associated Molecular Pattern) factors are precepted, and the defense signals also lead to PCD [17,20]. Thus, electrolyte leakage becomes a reliable measure of cell death and stress response. The early observation of electrolyte voltage from the inoculated cotyledons of Surpass400 and 01-23-2-1 implicated the early activation of defense response since the HR is found to trigger defense mechanisms including ion leakage, ROS signaling, hormonal signaling, etc. [17,31,37]. On the other hand, Westar samples started to have higher conductivity at 7 dpi, as suggested in Becker et al., 2017 [31], the RNA sequencing data revealed that the susceptible *B. napus* genotype triggered the same defense-related genes as the resistant genotype, however, the incompatible interaction activates the earlier expression of those genes compared with the compatible interaction, causing the different disease severity between susceptible and resistant genotypes.

Since *L. maculans* is hemibiotrophic, it undergoes the biotrophic stage first and then reaches the necrotrophic stage. Biotrophs usually exploit the nutrient from the living cells, it penetrates the plant cell wall and membrane with fungal structures such as haustoria and hyphae [44,45,46]. Evidence also showed that around the early stage of *L. maculans* infection upon *B. napus*, the fungus secretes cell wall degrading enzymes (CWDEs), and this physiological process is considered as one aspect of its pathogenicity [37,47]. Sexton et al. (2000) [37] reported that the highly virulent *L. maculans* races secrete the CWDEs at an early stage.

When blackleg fungus infects successfully, fungal hyphae develop in intercellular space during the biotrophic stage, and no obvious damage was made upon host cells [48]. Thus, early cell senescence becomes an effective strategy against biotrophic pathogens, to prevent further colonization and exploitation of host nutrients [16,45,48].

The early intensive diffusion of H_2_O_2_ from Surpass400 and 01-23-2-1 connect their resistant responses against the fungus, the accumulation of brownish discoloration (i.e., H_2_O_2_) around the origins of inoculation indicated a series of defensive responses including cell senescence from the host, which will hinder the further fungal growth. This may explain the similar pattern of cell death that occurred around the sites of inoculation, which was validated by trypan blue staining (TBS). The intermediate and resistant cotyledons tended to induce a protective region together with early hyphal development, so that the further intercellular penetration by the hyphae could be suppressed. The regional secretion of H_2_O_2_ and cell death were also found from other HR cases, this also accompanies other defense responses such as papillae development and cell wall alteration [21,49]. Moreover, the co-existence of regional cell death and H_2_O_2_ accumulation was also found from other examples of HR [50]; Nováková et al., (2015) [51] also suggested the potential function of H_2_O_2_ in restricting *L. maculans* development in *B. napus*. The findings from DAB and cell death assays revealed that the two types of incompatible interaction, intermediate (Surpass400–H75 8-1) and resistant (Surpass400–H77 7-2 and 01-23-2-1–H75 8-1/H77 7-2) were able to induce intense early (5 dpi) H_2_O_2_ accumulation and cell death as the priming defense to achieve effective defense against fungal proliferation on the plant tissues.

ROS generation and signaling play versatile roles in stress tolerance in the plant body. The superoxide (O_2_^−^) molecules are initially produced by NADPH oxidases or respiratory burst oxidase homologues (RBOH’s) and converted into hydrogen peroxide (H_2_O_2_) by superoxide dismutases (SOD’s) [14,34]. The stable and membrane-permeable properties make H_2_O_2_ molecules able to induce systemic responses against various biotic and abiotic stresses [12]. RBOHD and F are two the NADPH oxidases inducing ROS accumulation during plant defense response [52,53]. RBOHD and F are the two NADPH oxidases that have been well studied in *Arabidopsis thaliana* defense [30,52,53,54]. Calcium leakage, reactive oxygen intermediates (ROI) and peroxide were reduced in *rbohD*, *rbohF*, and *rbohD/rbohF* double mutants [53]. RBOHs are regarded as the central factors to trigger ROS signaling in plant cells [11,15,54]. RBOHD initiates the cell-to-cell ROS signaling which is called “ROS wave”, by transmitting H_2_O_2_ extracellularly. Evidence suggested that RBOHD was involved in early acute ROS signaling in defense and tolerance against various challenges [12,15], and RBOHD plays important roles in ROS production when the host recognizes the pathogen successfully on the site of infection [52]. *RBOHD* and *F* working together can fully activate basal resistance, the mutation of both genes abolishes ROS production and makes it easier for pathogens to infect [52,53]. As shown in Figure 4, the early expression of *RBOHD* from Surpass400 and 01-23-2-1 implied that this gene might also play crucial roles in *B. napus* such as in Arabidopsis during pathogenic infection. There are also some differences between the onset pattern between *RBOHD* and *F*, as such, *RBOHF* did not show high expression at 5 dpi in Surpass400, and 01-23-2-1 cotyledons were found to have the most pronounced up-regulation at 5 dpi while for *RBOHD*, the gene was up-regulated earlier at 1 dpi. These two genes were found to be regulated differently in *Arabidopsis thaliana*, and *RBOHD* plays more dominant roles in activation against pathogenic invasion [52]. It is also noted that the Westar genotype also displayed high expressions of both *RBOH* genes during necrotrophic stage (11 dpi). When infected, both compatible and incompatible interactions can trigger an oxidative burst. Therefore, it is normal to see massive a regulation of ROS-related genes when the plant tissue is heavily infected, however, the timing of the coordination of various regulators seems to be more important. By analyzing host-cell-wall-degrading enzymes (CWDEs) from the pathogen, Sexton et al., (2000) [37] implied that early restriction of fungal development is a crucial factor for *B. napus* cotyledon to achieve resistance towards *L. maculans*.

ROS signaling plays important roles in lesion development and cell senescence on plants, on the other hand, other factors such as JA signaling at the same time, can also attenuate to prevent the excessive damage by ROS [19,29]. Moreover, Becker et al., (2017) [31] also listed various types of genes which were activated from resistant *B. napus* genotype at 3 dpi. They include the factors in pathogen perception, callose deposition, sulfur metabolism, and lignification, whereas at the same time point, genes related to the negative regulation of plant defense and senescence were also activated. It seems that the resistant genotypes trigger massive signals from both up-regulation and down-regulation sides of defense at an early stage of infection, which hinders the fungal development during hyphal stage. On the other hand, during late stage (necrotrophic), the pathogen colonizes too widely. Thus, it is impossible for the host to achieve effective resistance, and the defense signals including ROS tend to express in a large amount to halt the further development, which produced such high levels of *RBOH* genes at 11 dpi in Westar.

Therefore, the high amount of the fungal cells pushed the host to evoke more defense signals to cope with the pressure of self-defense, similar to the cases in susceptible Westar and intermediate Surpass400–H75 8-1 after 7 dpi. Surpass400–H75 8-1, as the intermediate interaction, displayed both resistant and susceptible traits, as the samples taken from the inoculation pair both had the anticipated activation of *RBOHD* expression and H_2_O_2_ (same as resistant interaction), and the late induction of electrolyte leakage and *EDS1* expression (same as susceptible interaction).

On the other hand, the absence of co-expression between *PAD4* and *EDS1* was not expected. According to previous studies, the expression of *PAD4* is dependent upon *EDS1*, the interaction between EDS1 and PAD4 seems to enhance the HR by further SA accumulation [38,55]. In protein level, EDS1 and PAD4 interact each other, and trigger *R* gene-related resistance [38,39,46,56]. However, the function of *EDS1* is not totally dependent on *PAD4*, EDS1 is also able to dimerize itself (i.e., EDS1–EDS1 interaction) or bind with another PCD factor SENESCENCE ASSOCIATED GENE 101 (SAG101), moreover, those types of interaction contribute to innate immunity [38,39,55]. EDS1 itself also triggers partial *R* gene-related defense and SA accumulation [38,55]. Therefore, in this study, *EDS1* is highly expressed in defense response alone, without the cooperation with *PAD4*.

Finally, yet importantly, there was no strong trend of the early activation of *MPK3/6* from Surpass400 and 01-23-2-1. Besides gene expression, the function of MAPK factors is also related to phosphorylation, which activates downstream defense factors [42,57]. It is necessary to postulate that early ROS activation in *B. napus* might promote more on phosphorylation than expression.

## 4. Materials and Methods

### 4.1. Plant Materials

*Brassica napus* plants were grown in Sunshine Professional Growing Mix (SumGro Horticulture, Agawam, MA, USA), in 16 h of light (22 °C) (Photosynthetically Active Radiation (PAR) 300 μmole(m^−2^·s^−1^)) and 8 h dark (16 °C) at 50 to 60% relative humidity.

### 4.2. Pathogen Cultivation

*Leptosphaeria maculans* isolates were cultured on V8 juice (Campbell’s, Camden, NJ, USA) at room temperature under the fluorent tube light. The isolates were cultured for 10 to 14 days to obtain a sufficient amount of pycnidiospores. Each plate was scraped off and washed with 2 mL of distilled water to collect pycnidiospores and make inoculum stock solutions. The stock solutions were stored at −20 °C.

### 4.3. Pathogen Inoculation

Two L. maculans isolates were selected for inoculation: HCRT75 8-1 (Genotype: avrLm1, AvrLm2, avrLm3, avrLm4, AvrLmJ1-5, AvrLm7, AvrLm6, avrLm9, AvrLm11, avrLepR1 and AvrLepR2) and HCRT77 7-2 (Genotype: AvrLm1, avrLm2, avrLm3, AvrLm4, AvrLmJ1-5, AvrLm7, AvrLm6, avrLm9, AvrLm11, avrLepR1 and avrLepR2).

Three *B. napus* genotypes were selected to be inoculated: Westar (no *Rlm* gene), Surpass400 (*BLMR1/LepR3* and *BLMR2/RlmS*), and 01-23-2-1 (*Rlm7*).

The cotyledons of *B. napus* cultivars were inoculated 7 days after sowing (seedling stage) by puncture inoculation. Each lobe of cotyledons was punctured by a sterile needle twice from each side, to have 4 inoculation points on each seedling of the canola plant.

Two selected *L. maculans* isolates (HCRT75 8-1 and HCRT77 7-2) produced three distinct levels of severity on three *B. napus* cultivars (Westar, Surpass400, and 01-23-2-1). The genotype Westar without any *Rlm* genes produced susceptible phenotypes with both isolates, while Surpass400 (*Rlm* genes: *BLMR1/LepR3* and *BLMR2/RlmS*) exhibited some level of resistance on both isolates, as such, intermediate towards HCRT75 8-1 and resistant (hypersensitive response, HR) towards HCRT77 7-2. Finally, the cultivar 01-23-2-1 (*Rlm* genes: *Rlm7*) showed typical HR resistance on both isolates (Figure 1).

### 4.4. Electrolyte Leakage Measurement

The cotyledons (6 cotyledons from 3 biological replicates) were cut into small leaf disks (round, 5 mm in diameter) with the cork borer. The leaf disks were washed for 30 min in 10 mL ultrapure water and transferred into another round of fresh ultrapure water (25 mL). After 5 h, the electrolyte leakage was measured in voltage from the soaked ultrapure water by the VWR sympHony conductivity meter (Radnor, PA, United States).

### 4.5. 3,3′-Diaminobenzidine (DAB) Staining

The DAB staining solution was prepared by dissolving 40 mg of DAB (Sigma-Aldrich, St. Louis, MO, USA) in 200 μL of dimethylformamide in 40 mL of water. Cotyledons were soaked in the staining solution in the dark and shaken overnight. The stained cotyledons were discolored by 95% ethanol. The experiment is followed by the protocol of Sašek et al. (2012) [6].

### 4.6. Trypan Blue Staining (TBS)

The trypan blue stock solution was prepared by mixing 10 mL of phenol, 10 mL of glycerol, 10 mL of lactic acid, 10 mL of water, and 0.02 g of trypan blue powder (Sigma-Aldrich, St. Louis, MO, USA). The working solution was prepared by dissolving the stock solution with ethanol (96%; 1:2, *v*/*v*). *B. napus* cotyledons were soaked in the working solution and boiled in a water bath for 1 min, incubated in the solution overnight and washed in chloral hydrate solution (2.5 g of choral hydrate in 1 mL of distilled water).

### 4.7. Gene Expression Analysis

Frozen cotyledons (12 cotyledons, 6 seedlings, 3 biological replicates) were ground in liquid nitrogen using pestles and mortars. Total RNA was extracted with TRI reagent (Sigma-Aldrich, St. Louis, MO, USA). Total RNA was purified by DNase I treatment with DNase I recombinant, RNase-free kit (Roche). Purified RNA was used to synthesize cDNA with the GOScript Reverse Transcription System (Promega). The cDNA stock solution was diluted into a concentration of 100 ng/µL. The quantitative-PCR was performed by mixing 1 µL of cDNA (100 ng) into the 10 µL reaction system of IQ^TM^ SYBR^®^ Green Supermix (BioRad, Hercules, CA, USA).

The qPCR program used for all analyzed genes was: 95 °C for 3 min; followed by 39 cycles of 95 °C for 15 sec, and 60 °C for 20 sec; followed by a melting curve analysis.

All qPCR primers are listed in Supplementary Table S1. The relative level of gene expression was analyzed with the 2^−ΔΔCT^ method described by Livak and Schmittgen, (2001) [58]. The expression of the studied genes was normalized by the house-keeping gene *ACTIN* (NM_001316010.1).

### 4.8. Statistical Analysis

Unless specified, the analyses of samples used at least three biological replicates. The statistical analyses were performed using the Fisher’s Least Significant Difference (LSD) method with the SAS 9.4 software (SAS Institute, Cary, NC, USA) [59]. The Fisher’s LSD was applied to gene expression and electrolyte leakage measurement, in order to observe the effectiveness of resistance in the three genotypes when inoculated with two isolates.

## 5. Conclusions

The data from this article revealed that ROS metabolism and signaling played potential roles in the host-microbe interaction in the *B. napus*–*L. maculans* pathosystem. Intermediate and resistant genotypes displayed intense hydrogen peroxide (H_2_O_2_) diffusion and cell death around the site of inoculation. Moreover, ROS/PCD-responsive genes tended to express earlier in the intermediate and incompatible interactions. Those findings suggested that earlier activation of ROS-related defense mechanisms is an essential component of effective resistance in *B. napus* against *L. maculans*.

## Data Availability

The original contributions presented in the study are included in the article/supplementary materials, further inquiries can be directed to the corresponding author/s.

## Supplementary Materials

The following are available online at https://www.mdpi.com/article/10.3390/ijms22094812/s1.
